# Risk factors for child maltreatment by the utilization of medical service and socioeconomic environment in Taiwan

**DOI:** 10.1097/MD.0000000000013728

**Published:** 2018-12-28

**Authors:** Yi-Chen Hsin, Yu-Ching Chang, En-Pei Lee, Cheng-Hsun Chiu, I.-.Jun Chou, Shao-Hsuan Hsia, Kuang-Lin Lin, Jung Lee, Jing-Long Huang, Chao-Jan Wang, Han-Ping Wu

**Affiliations:** a Division of Pediatric General Medicine, Department of Pediatrics, Chang Gung Memorial Hospital at Linko, Kweishan; b College of Medicine, Chang Gung University; c Division of Pediatric Critical Care Medicine; d Division of Pediatric Infectious Diseases; e Division of Pediatric Neurology; f Division of Pediatric Allery, Asthma, and Rheumatology, Department of Pediatrics; g Department of Medical Imaging and Intervention, Chang Gung Memorial Hospital at Linko, Kweishan, Taoyuan; h Department of Pediatric Emergency Medicine; i Department of Medical Research, Children's Hospital; j Department of Medicine, College of Medicine, China Medical University, Taichung; k Chang Gung Memorial Hospital Study Group for Prevention and Protection Against Child Abuse and Neglect, Chang Gung Memorial Hospital at Linko, Kweishan, Taoyuan, Taiwan.

**Keywords:** child abuse, child maltreatment, neglect, physical abuse, sexual abuse

## Abstract

Child maltreatment is complicated by cultural, welfare, and socioeconomic factors. However, the relationship between child maltreatment and socioeconomic factors has not been completely understood. We investigated risk factors for child abuse and neglect in Taiwan.

The data in our study was obtained from Taiwan National Statistics at county level from 2004 to 2015. We included 4 areas (eastern, western, southern, northern) involving 20 cities and counties. The trends of child maltreatment rate based on different years and different areas were surveyed. In addition, panel data analysis was used to analyze the links between child maltreatment rate and socioeconomic factors.

An increasing trend of child maltreatment rate in Taiwan was observed. During the past decade, child maltreatment rate increased from 14.5 in 2004 to 23.4 cases per 10000 children in 2014. The peak, which was 43 cases per 10000 children, occurred in 2012. Significant geographical differences were observed, and the highest child maltreatment rate was seen in eastern Taiwan. Panel data analysis revealed a lag effect of the unemployment rate on child maltreatment rate at the county level: the child maltreatment rate increased by 7 percent, while the prior unemployment rate increased by one percent. In addition, the medical personnel density was related to the child maltreatment rate within the county.

Previous unemployment rate had a lag impact on child maltreatment occurrence. Unemployment rate has not only a direct impact on the economy but also sequential effects on child maltreatment.

## 1 Introduction

Child maltreatment is a critical public health issue and comprises physical, sexual, or emotional abuse and/or neglect of children. It is growing as a global phenomenon across low- and high-income countries.^[1]^ The increase in child maltreatment has gained more attention recently. In the United States, victims of child maltreatment increased by 3.8 percent from 2011 to 2015. In 2015, there were 4 million referrals to the child protective services (CPS) agencies, alleging maltreatment, involving more than 7 million children in the United States. The child maltreatment rate in the United States in 2014 was approximately 9.4 per thousand.^[2]^ The World Health Organization (WHO) reported that possibly more than 41000 children, younger than 15 years, died of homicide every year worldwide. Child maltreatment either results in premature deaths or leads to adverse childhood experiences in survivors, who develop long-term physical, psychological, and behavioral sequelae.^[3–6]^ Child abuse and neglect have turned out be a huge burden to the whole society, as costly as any other major public health problem. The total lifetime financial costs of new child maltreatment cases in the United States in 2008 was estimated to be about 124 billion United States Dollars.^[7]^ Thus, action against child maltreatment will not only ensure the well-being of children but also have positive financial implications.

Child maltreatment is multifactorial involving several aspects such as culture, biology, society, economy, and environment. To solve such a complicated problem, ecological studies may provide a good point of view to the researchers and policy makers. The influence of poverty, unemployment, and financial depression on the occurrence of child maltreatment has been discussed in western countries.^[8–11]^ However, little is known about the impact of socioeconomic changes on child maltreatment rate in other parts of the world, such as in Taiwan. Utilization of medical services has rarely been discussed in the literature. Therefore, we investigated the risk factors for child abuse and neglect and analyzed the impact on child maltreatment rate.

## 2 Materials and methods

Our data were obtained from the Taiwan National Statistics at the county level from 2004 to 2015. In our study, we included 4 areas (eastern, western, southern, northern) involving 20 counties (Northern area: Taipei city, New Taipei city, Taoyuan city, Yilan county, Hsinchu county, Keelung city, Hsinchu city; Southern area: Tainan city, Kaohsiung city, Chiayi county, Pingtung county, Penghu county, Chiayi city; Middle area: Taichung city, Miaoli county, Changhua county, Nantou county, Yunlin county; Eastern area: Taitung county, Hualien county). Child maltreatment rate was defined as substantiated cases of child maltreatment per 10000 children under the age of 18. Substantiated cases indicated those confirmed by detailed studies and who had received intervention, following referral or reporting.

Child maltreatment rates in Taiwan and at the county level from 2004 to 2015 were presented graphically. Descriptive statistical analysis and repeated-measures analysis of variance (RM-ANOVA) were used to compare child maltreatment rates across Taiwan. The RM-ANOVA is widely used in statistical analysis to compare the mean values of the study outcome variable across different time points or between groups that are repeatedly measured. Therefore, we used RM-ANOVA in our study to compare child maltreatment rates across 4 areas of Taiwan. The panel data analysis deals with 2-dimensional data over time and over counties, so the panel data analysis was adopted to examine the impact of changes in socioeconomic environment and utilization of medical services on child maltreatment rate. Thus, we used panel data analysis to analyze the data of county-level from 2004 to 2015. The Hausman test was used to select the fixed-effect or random-effect model for panel data analysis. Fixed effect model was chosen in our study according to the result of Hausman test. The dependent variable was county-level child maltreatment rate (substantiated cases per 10000 children). The independent variables in our study contained county-level unemployment rate, low-income rate, and social worker density, which represent the socioeconomic environment; as well as the density of medical personnel, which represent utilization of medical services. Interactions between area and utilization of medical services were also examined. In the descriptive analysis, values were presented as mean ± standard deviation (SDs). Statistical significance was set at *P* <.05. All statistical analyses were conducted using STATA, version 13.0 (Stata Corp., College Station, TX).

## 3 Results

**Figure 1:**
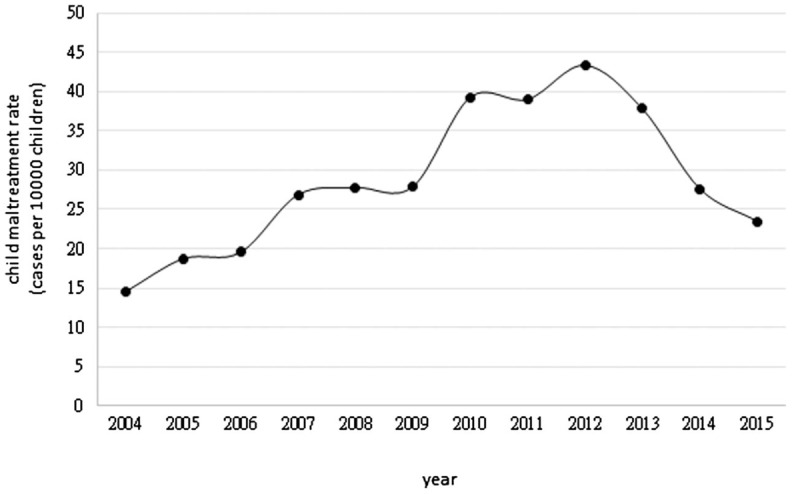
Trend of child maltreatment rate in Taiwan from 2004 to 2015.

**Figure 2:**
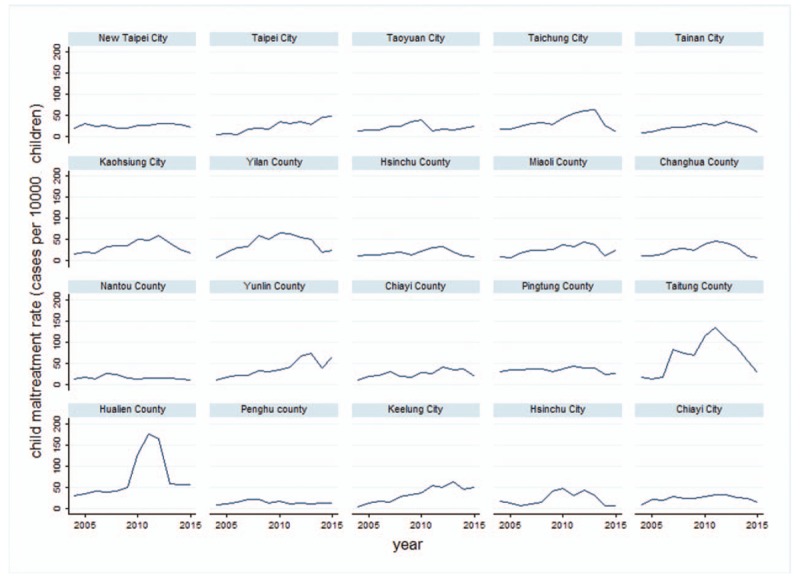
Child maltreatment rate by county from 2004 to 2015.

There were 161,183 substantiated cases of child maltreatment in Taiwan from 2004 to 2015 with an average of 13,431 cases per year. During the study period, we found that the trend of child maltreatment rate was growing. Figure 1 shows the increasing trend in the child maltreatment rate in Taiwan from 2004 to 2012, but a slight descent is observed from 2013 onwards. During the past decade, child maltreatment rate increased from 14 cases per 10,000 children in 2004 to 23 per 10,000 in 2015. The peak, which was 43 cases per 10,000 children, occurred in 2012. We also surveyed the child maltreatment rate by county from 2004 to 2015, represented in Figure 2. The curves in most counties show a gradual upward trend, but 2 humps are obviously observed in Taitung county and Hualien county, both located in eastern Taiwan. The distribution of child maltreatment rate has significant geographical differences and the highest child maltreatment rate is observed in eastern Taiwan (Table 1).

**Table 1:**
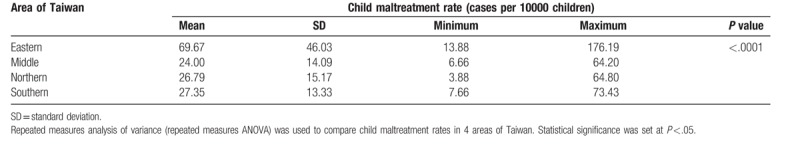
Child maltreatment rate in 4 areas of Taiwan.

**Figure 3:**
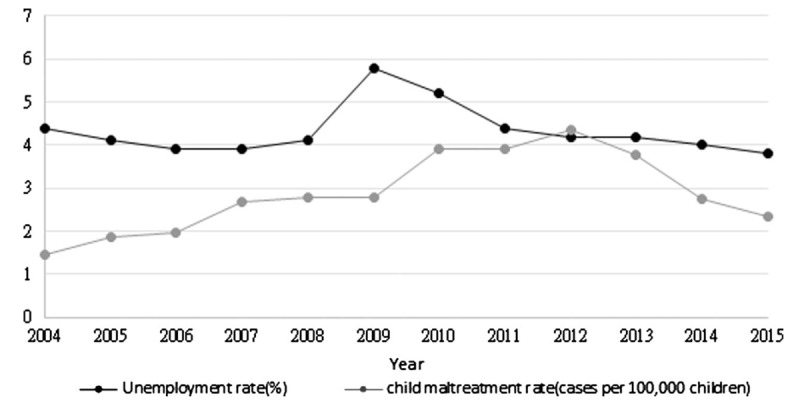
Unemployment rate and child maltreatment rate in Taiwan from 2004 to 2015.

The results of panel data analysis revealed that the unemployment rate had a lag effect on the child maltreatment rate. At the county level, child maltreatment reported rate increased to 7 cases per 10,000 children while the previous unemployment rate increased by 1 percent (*P* = .001). The current unemployment rate did not significantly influence the child maltreatment rate. Figure 3 displays the unemployment rate and child maltreatment rate (cases in 100,000 children) in Taiwan. Based on the panel data analysis, low-income rates and density of social workers were not associated with child maltreatment rate within counties. The county-level medical personnel density, as a measure of the utilization of medical services, was, however, related to the county-level child maltreatment rate. Within counties, the medical personnel density had a much smaller impact on child maltreatment rate compared to the previous unemployment rate. A 1 percentage point increase in medical personnel density, increased the child maltreatment rate by 0.45 (*P* <.05). When we examined the interaction between areas and density of medical personnel, the interaction between the eastern area and medical personnel density was amplified nearly 3-fold (Table 2).

**Table 2:**
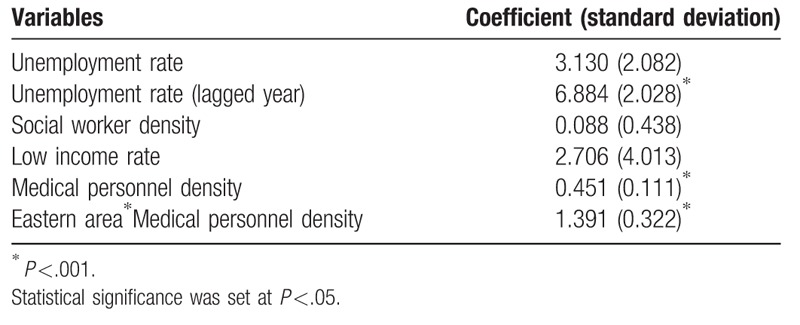
Fixed effect estimates for county-level child maltreatment rate from 2004 to 2015.

## 4 Discussion

Our study provided a comprehensive view toward child maltreatment in Taiwan from 2004 to 2015. Our findings made contribution to current knowledge. First, the unemployment rate of prior year was positively associated with the child maltreatment rate at county level. Second, medical personnel density was associated with child maltreatment rate at county level, particularly in eastern Taiwan. These finding will contribute to further policy adjustment to reduce child maltreatment in Taiwan.

There is evidence to show that poverty is related to child maltreatment. As we know, income inequality, low income, and unemployment worsen poverty. In the United States, the income inequality did influence child maltreatment rates. The county-level Gini index increased and the child maltreatment rate rose.^[12]^ However, in our study, low-income rate did not demonstrate a significant impact on the child maltreatment rate at county level in Taiwan. In addition, it is controversial whether the unemployment rate influences the child maltreatment rate. The association between unemployment rate and child maltreatment differs from state to state based on county-level data in the United States. In many counties of California, they found that unemployment rate was not related to the rate of out-of-home child maltreatment cases per 1000 children.^[13]^ The unemployment rate increased and medium-to-high risk reports of child maltreatment increased in Pennsylvania.^[14]^ Another study in New York suggests that increases in unemployment rates reduced child maltreatment rates, especially in metropolitan counties.^[10]^ In Taiwan, we found the unemployment rate had a delayed effect on the child maltreatment rate: the unemployment rate increased and the child maltreatment rate of the next year increased.

Our results using county-level data in Taiwan showed unemployment rate had a lag effect on child maltreatment rate. The labor and employment insurance in Taiwan provides 6 to 9 months of unemployment benefits to insured persons. The supplements might help those laborers who lost their job, to overcome the first few months. However, the situation might be more difficult for these families after 6 months. The families might suffer more stress either from persistent unemployment or from newly gained employment in the next year. That might explain why the child maltreatment rate increased following the unemployment rate of the previous year, but not the current year. Our findings point out, that policies assisting unemployed people and their families need to cover a longer period, possibly 12 months or more. Economic support could be provided through initiatives such as unemployment benefits, assistance to low-income families, and earned income tax credit (EITC).^[15]^ More social safety and family support to ensure child well-being, such as food banks and affordable childcare centers might alleviate the burden of these families. It is necessary to put in more effort in preventing child maltreatment, as the unemployment rate rises.

There are striking geographic variations in the child maltreatment rate in Taiwan. The eastern region of Taiwan had a higher rate of child maltreatment than the non-eastern region. The lowest population density, lowest hospital density, and highest divorce rate were also noted here. Aboringines is the main race of the eastern Taiwan. Racial disparities and multiple factors contributed to child maltreatment occurrence.^[16]^ Although there has been a universal National Health Insurance in Taiwan, an unequal utilization of medical services between the eastern and non-eastern regions of Taiwan exists. The medical personnel density was associated with child maltreatment rate at the county level. The impact of medical personnel density on child maltreatment rate was 3 times larger in eastern Taiwan. This finding implies that medical personnel play an important role in discovering child maltreatment, particularly in eastern Taiwan, although there are barriers to health care. The social worker density was not related to the child maltreatment rate within counties in Taiwan. The average caseload of social workers in Taiwan may be about 50 victims per social worker. The caseload of social workers in Taiwan may be much heavier than that in the United States.^[17]^ The case overload of social workers worsens the shortage of social workers.

The importance of our study is to demonstrate the key socioeconomic factors on child maltreatment rate in Taiwan. Our research took a broad view of the child maltreatment in Taiwan to provide a direction toward the creation of more comprehensive policies against child maltreatment. These ecological associations enable countries to better allocate limited resources to child protection programs.

Nevertheless, this study has some limitations. The retrospective study was conducted only in Taiwan and different areas may have different findings, leading to different outcomes. Another limitation was child maltreatment rate in our study was drawn from Taiwan National Statistics and based on the “substantiated” child maltreatment cases. As child maltreatment is under-reported, we are not sure that our finding for reported cases would be consistent with those unreported cases. Further studies may be needed to confirm our findings.

## 5 Conclusions

Our findings suggest that previous unemployment rate shows a lag impact on child maltreatment occurrence. The rise in the unemployment rate not only causes a direct impact on the economy but also has sequential effects on the child maltreatment rate.

## Author contributions

YCH analyzed and interpreted the data as well as drafted the manuscript; YCC and EPL reviewed the medical records, and analyzed the data. CHC, IJC and SHH reviewed the medical records and interpreted the data. JL, JLH and KLL interpreted the data. HPW and CJW designed and oversaw the study, interpreted the data, and revised the manuscript. All authors have read and approved the final manuscript for publication.

**Data curation:** Yu-Ching Chang, En-Pei Lee, PCHAN Study Group.

**Investigation:** Cheng-Hsun Chiu, I-Jun Chou, Shao-Hsuan Hsia.

**Methodology:** Kuang-Lin Lin.

**Resources:** Jung Lee, Jing-Long Huang.

**Supervision:** Chao-Jan Wang.

**Writing – original draft:** Yi-Chen Hsin.

**Writing – review & editing:** Chao-Jan Wang, Han-Ping Wu.
